# Comparison of mental health presentations of 16–25-year-olds to the Emergency Department during the COVID-19 pandemic.

**DOI:** 10.1192/j.eurpsy.2023.493

**Published:** 2023-07-19

**Authors:** C. Mohan, O. Conaty, A. Moughty, A. Doherty, E. Barrett, A. M. Clarke

**Affiliations:** ^1^Liaison Psychiatry, Mater Misericordiae University Hospital; ^2^Liaison Psychiatry, St.Vincent’s University Hospital; ^3^General Adult Psychiatry; ^4^Emergency Medicine, Mater Misericordiae University Hospital; ^5^Child & Adolescent Liaison Psychiatry, Children’s Health Ireland at Temple Street, Dublin, Ireland

## Abstract

**Introduction:**

Studies reported an initial decrease in the number of presentations and incidence of self-harm in young people during the pandemic. As the pandemic progressed young people may have experienced increased levels of distress, contributing to worsened mental health. There is a need for mental health services to evaluate the presentations of young people presenting to the Emergency Department (ED) so that services can meet the needs of young people.

**Objectives:**

To examine the mental health presentations of young people (aged 16-25) to the ED and how this may have changed since the start of the pandemic.

**Methods:**

This study reviewed all 16–25-year-olds presenting to the Mater Misericordiae University Hospital (MMUH) who were triaged with a mental health issue in a 2 month period (September-October) over three years – 2019 (Period A), 2020 (Period B), 2021 (Period C). Approval for this service evaluation was granted by the Clinical Audit and Effectiveness Committee at the MMUH.

**Results:**

Of 232 presentations across all periods, there was no significant difference in the number of presentations in each study period - Period A (n=76), Period B (n=79) and Period C (n=77). In all three periods, most presentations occurred out of hours (A: 57.9% [n=44]; B: 74.7% [n=59]; C: 68.8% [n=53]) statistically significant (p=0.034) from before (A) to during the pandemic (B and C). Out of hours arrival by ambulance was most common in in Periods A and B (45.5% and 55.9%). Over all three periods discharge home was the most frequent outcome of assessment (A: 69.7% [n=53]; B: 70.9% [n=56]; C: 76.6% [n=59]). Overall, there was a decrease in self-harm presentations over the period (A: 47.4% [n=36]; B: 41.8% [n=33]; C: 40.3% [n=31]). The percentage of presentations with self-laceration increased during the pandemic (A: 33.3% [n=12]; B: 39.4% [n=13]; C: 48.4% [n=15]). There was a significant increase in attendees who were already taking psychotropic medications (p<0.001).

**Conclusions:**

The findings suggest that the majority of 16–25-year-olds present out of hours and do not require admission. Although the number of presentations remained similar, the increase in out of hours presentations and arrivals by ambulance in Period B may reflect increased distress in the initial stages of the pandemic, and restricted access to services. The higher rates of medication prescribing suggests that these young people are already receiving health care, but that their needs are not being fully met. Mental health services should be designed to provide access to mental health care out of hours when young people are most likely to require them.

**Disclosure of Interest:**

None Declared

